# Tacrolimus Concentration Is Effectively Predicted Using Combined Clinical and Genetic Factors in the Perioperative Period of Kidney Transplantation and Associated with Acute Rejection

**DOI:** 10.1155/2022/3129389

**Published:** 2022-09-09

**Authors:** Fang Cheng, Qiang Li, Zheng Cui, Zhendi Wang, Fang Zeng, Yu Zhang

**Affiliations:** ^1^Department of Pharmacy, Union Hospital, Tongji Medical College, Huazhong University of Science and Technology, Wuhan 430022, China; ^2^Hubei Province Clinical Research Center for Precision Medicine for Critical Illness, Wuhan 430022, China; ^3^Department of Urology, Union Hospital, Tongji Medical College, Huazhong University of Science and Technology, Wuhan 430022, China

## Abstract

**Background:**

Tacrolimus has unpredictable pharmacokinetic (PK) characteristics, which are partially attributed to CYP3A5 polymorphism. The potential effects of clinical factors in the postoperative period of transplantation on tacrolimus PK and those of early tacrolimus PK variability on clinical outcomes are yet to be clarified.

**Methods:**

We examined the genetic and clinical factors affecting early tacrolimus PK variability in 256 kidney transplant recipients. The relationships among tacrolimus exposure, graft function delay (DGF), and acute rejection (AR) were further explored. *Findings*. The CYP3A5 genotype were strongly associated with tacrolimus concentration/dose ratio (*C*_0_/*D*). Additionally, ABCB1 (rs1045642 and rs2032582) and ABCC2 (rs3740066) were found to have potential independent effects on early tacrolimus *C*_0_/*D* in multivariate analysis. Red blood counts and albumin level were the most significant clinical factors associated with tacrolimus *C*_0_/*D*. Wuzhi capsule also exerted an effect on tacrolimus PK. A model combined with pharmacogenetic and clinical factors explained 43.4% tacrolimus PK variability compared with 16.3% on the basis of CYP3A5 genotype only. Notably, increasing tacrolimus concentrations in the early postoperative stage were associated with AR, but not DGF.

**Conclusions:**

Combined analysis of genotype and specific clinical factors is important for the formulation of precise tacrolimus dose regimens in the early stage after kidney transplantation.

## 1. Introduction

Tacrolimus is an important component of immunosuppression therapy after solid organ transplantation [1]. However, treatment with tacrolimus is associated with a number of significant disadvantages, such as narrow therapeutic index, various side-effects, and significant individual differences [2, 3]. Therefore, therapeutic drug monitoring (TDM) is widely used to adjust the tacrolimus dose for reducing the risk of toxicity and acute rejection in individual transplant patients. However, the target tacrolimus concentration may lag behind owing to the differential first-pass effects among individuals, leading to reduce treatment effect or increase adverse reactions.

Genetic polymorphisms play a critical role in tacrolimus pharmacokinetic (PK) variability [4]. Tacrolimus is mainly metabolized by cytochrome P4503A5 (CYP3A5) enzymes in the liver [5]. CYP3A5 genetic polymorphism is thus a main contributory factor to tacrolimus PK variability. The effect of CYP3A5 genotype on tacrolimus metabolism is well established in previous studies [6, 7]. The study conducted by Chen and Prasad [8] showed that CYP3A5 expressers with at least one CYP3A5∗1 allele require 50% higher tacrolimus doses compared to CYP3A5 nonexpressers with homozygous CYP3A5∗3, CYP3A5∗7, or CYP3A5∗6 alleles in different populations. Furthermore, CYP3A5 polymorphisms are proposed to explain 40–50% of tacrolimus PK variability [9]. Several guidelines to date have recommended a CYP3A5 gene-guided tacrolimus dosing regimen in kidney transplantation patients [10, 11]. However, Shuker and coworkers demonstrated limited effectiveness of CYP3A5-guided dose adjustment in reaching the target concentration range in kidney transplant recipients [12], suggesting that additional factors play an important role in tacrolimus PK variability. Recently, correlations of ABCB1, CYP3A4, ABCC2, POR, and PXR gene variants with tacrolimus PK have been reported in different transplant populations [13–15]. Previous pharmacogenetic and clinical studies on predictors of tacrolimus metabolism have focused on a wide range of times, from months to years after kidney transplantation [16, 17]. The clinical reality is that patient' condition, clinical status, and medications often change rapidly during the perioperative period of kidney transplantation, resulting in pronounced tacrolimus PK variability. Furthermore, patients may be particularly vulnerable to adverse effects during this time.

Tacrolimus PK variability within the early posttransplant period is associated with poor outcomes after kidney transplantation [18]. Delayed graft function (DGF), a manifestation of acute graft injury, may be improved by slow tacrolimus metabolism [19, 20]. Acute rejection (AR) is an immune-mediated allograft injury potentially caused by low tacrolimus concentrations [21]. Both DGF and AR are closely related to death, graft dysfunction, and poorer outcomes [22, 23]. Elucidation of the predictors of early tacrolimus PK variability may therefore be key to informing effective precision dosing strategies. Furthermore, to determine clinical utility and justify the expense and effort of pharmacogenetic dosing, the effects of early tacrolimus concentrations on DGF and AR rates need to be established. To clarify this issue, the factors influencing early tacrolimus PK variability and relationships among early tacrolimus concentrations, DGF, and AR after kidney transplant were explored in this study.

## 2. Materials and Methods

### 2.1. Patients Selection

This study included 256 kidney transplantation patients from January 2015 to December 2019 in Union hospital, Tongji Medical College, Huazhong University of Science and Technology. The enrollment criteria were as follows: patients receiving conventional tacrolimus-based immunosuppressive regimen (tacrolimus, mycophenolate mofetil, and corticosteroids) and age over 18 years. The exclusion criteria were as follows: patients' age less than 18 years, combined other organ transplantation, receiving cyclosporine or intravenous tacrolimus, treatment with drugs affecting tacrolimus metabolism (such as diltiazem, ketoconazole, berberine, and voriconazole), or lack of relevant data. Our study was approved by the Institutional Ethics Board of Tongji Medical College, Huazhong University of Science and Technology (Wuhan, China) ([2018] S331). This was a retrospective analysis and did not interfere with patient diagnosis or the treatment process. The patients in our study received kidney transplant from donations after cardiac death (DCD). DCD procedures were performed by the Organ Procurement Organization of Union Hospital, Tongji Medical College, Huazhong University of Science and Technology, in accordance with the Declaration of Istanbul. Data on demographic, laboratory tests, basic diseases, and medication in the perioperation period were obtained through the electronic medical record system in our hospital.

### 2.2. Immunosuppressive Regimens

All patients were administered a tacrolimus-based triple immunosuppressive regimen. Tacrolimus was taken orally on the second day after transplantation, with an initial dose of 3.0-5.0 mg, twice a day. Mycophenolate mofetil was administered twice a day (0.5-1.0 g) on the day of transplantation. All patients were administered with methylprednisolone (500 mg daily) intravenously three days after kidney transplantation and 60 mg daily oral methylprednisolone from day 4, which was gradually reduced to a maintenance dose (20 mg daily). In cases of acute rejection, rabbit anti-human thymocyte immunoglobulin or anti-human T cell rabbit immunoglobulin was administered for 3-7 days, or methylprednisolone pulse therapy was performed.

### 2.3. Therapeutic Drug Monitoring

Tacrolimus was administered on day 2 posttransplantation, and the tacrolimus concentration was measured on day 5. The tacrolimus concentrations were measured three times weekly during the perioperative period. In cases where rejection or adverse reactions were suspected, the measurement frequency was higher. A Roche Cobas ® E411 electrochemiluminescence analyzer was used to measure tacrolimus concentration in whole blood. In our hospital, the target concentration range is 8-12 ng/mL during the perioperative period. Tacrolimus PK variability was quantified based on the tacrolimus concentration/dose ratio (*C*_0_/*D*) [24], in keeping with numerous previous studies.

### 2.4. Genotypes

Peripheral blood samples were used for genotyping with the Capital Biotechnology Precision Medicine Research Array (CBT-PMRA) kit (Thermo Fisher Scientific, Waltham, MA, USA) on the Applied Biosystems Axiom 2.0 platform. The SNPs reported to potentially affect tacrolimus PK were selected, including CYP3A4∗22 (rs35599367), CYP3A5∗3 (rs776746), CYP3A4∗1B (rs2740574), ABCC2 (rs2273697, rs3740066 and rs717620), ABCB1(rs2032582, rs1045642, and rs1128503), PXR (rs6785049), and POR∗28 (rs1057868).

### 2.5. Clinical Outcomes

In our study, DGF was defined as hemodialysis within seven days posttransplant and AR as an acute deterioration of kidney function associated with specific pathologic changes in graft biopsies, occurring in the first year after kidney transplant.

### 2.6. Statistical Analysis

All statistical analyses were performed with SPSS V.24.0 software. When continuous variables were normally distributed, data were expressed as the mean ± standard deviation. Otherwise, continuous variables were described as median and interquartile range (IQR). Categorical variables were presented in frequency and percentage. The effects of pharmacogenetic and clinical variables on tacrolimus *C*_0_/*D* were modeled via linear mixed effect regression. In order to avoid the loss of independent influencing variables (no significant difference in the univariate analysis due to the influence of other confounding factors), we selected variables with *p* value < 0.4 in the univariate analysis and clinically significant variables for stepwise regression to obtain the final multivariate model. We established two models: (1) including CYP3A4 genotype only and (2) incorporating both clinical variables and genotype factors. The associations between tacrolimus concentration and DGF and AR were evaluated with the *χ*^2^ test. *p* value < 0.05 was considered significantly different.

## 3. Results

### 3.1. Patient Characteristics

Among the 256 patients, 178 (69.5%) were male. Demographic and clinical data are shown in Table 1. The median age of patients was 41 years (IQR: 34−50 years), and age range was 19-65 years. Hypertension (*N* = 187 [73.0%]) and anemia (*N* = 117 [45.7%]) were the most common basic diseases in kidney transplant recipients. Overall, 224 (87.5%) patients received induction therapy with antithymocyte globulin, and 32 (12.5%) received basiliximab therapy. An immunosuppressive regimen with mycophenolate mofetil as an antiproliferative agent was administered to 239 (93.4%) of the patients. All recipients underwent transplantation for the first time, using DCD as the source of kidneys.

### 3.2. Pharmacogenetic Analysis

We examined the effects of CYP3A4∗1B (rs2740574), CYP3A5∗3 (rs776746), CYP3A4∗22 (rs35599367), ABCC2 (rs2273697, rs3740066 and rs717620), ABCB1 (rs2032582, rs1045642, and rs1128503), PXR (rs6785049), and POR∗28 (rs1057868) polymorphisms on early tacrolimus *C*_0_/*D* after kidney transplantation. Notably, CYP3A4∗1B mutations were absent, and only two among the 256 patients contained the CYP3A4∗22 mutation. Accordingly, these two genotypes were excluded from follow-up analysis.

**Figure 1:**
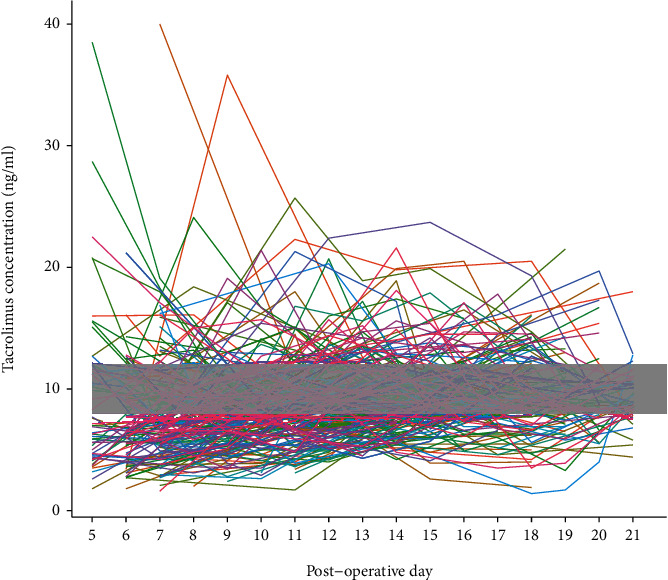
Individual trends of tacrolimus concentration.

**Figure 2:**
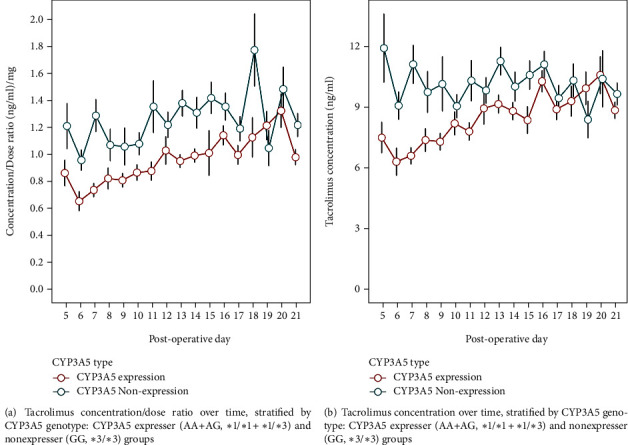
Effect of the CYP3A5 genotype on tacrolimus PK variability.

The individual trends of early tacrolimus concentrations in all patients after transplantation are shown in Figure 1, which greatly deviated from the target concentration range of 8-12 ng/mL. The target range was reached in 64 (25.0%) patients during the 3-week postoperative period. We assigned the 256 patients into two groups: CYP3A5 expressers (AA+AG, ∗1/∗1+∗1/∗3) and nonexpressers (GG, ∗3/∗3). The tacrolimus concentrations and *C*_0_/*D* in the CYP3A5 nonexpresser group were significantly higher than those in the CYP3A5 expresser group, indicating a strong association between CYP3A5 genotype and tacrolimus metabolism (Figure 2). However, no significant differences were evident among ABCB1, ABCC2, PXR, or POR∗28 alleles and tacrolimus PK in the univariate analysis (Figure S1).

### 3.3. Prognostic Factors of Early Tacrolimus *C*_0_/*D* after Transplantation

The results of univariate analysis are shown in Table S1, and the multivariable mixed effects model is presented in Table 2. A strong correlation between tacrolimus *C*_0_/*D* and CYP3A5 genotypes was further validated in the multivariable mixed effects model. After eliminating the effect of other confounding factors by multivariate analysis, ABCB1 (rs1045642, and rs2032582) and ABCC2 (rs3740066) were found to have potential independent effects on early tacrolimus *C*_0_/*D*. Compared with ABCB1 (rs1045642) TT genotype carriers, tacrolimus *C*_0_/*D* in patients with CC and CT genotypes was decreased by 15.03% and 14.59%, respectively. Patients with ABCB1 (rs2032582) AA genotype carriers increased tacrolimus *C*_0_/*D* by 16.52% compared with GG genotype carriers. In addition, tacrolimus *C*_0_/*D* increased by 14.42% in patients with ABCC2 (rs3740066) TT genotype carriers compared with CC carriers. Several clinical variables additionally showed significant associations with tacrolimus *C*_0_/*D*, including red blood counts (RBC), albumin (ALB), and Wuzhi capsule (WZC). RBC, ALB, and WZC were associated with 22.17%, 2.19%, and 27.2% increase in tacrolimus *C*_0_/*D*, respectively. A model incorporating combined genetic and clinical factors accounted for 43.4% (*R*^2^ = 0.434) of tacrolimus PK variability compared with 16.3% (*R*^2^ = 0.163) for CYP3A5 genotype status only.

### 3.4. Clinical Outcomes

**Figure 3:**
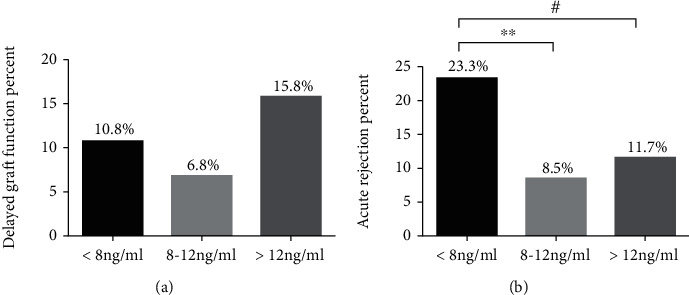
Associations among early tacrolimus concentration, delayed graft function (DGF), and acute rejection (AR). The average tacrolimus concentration was classified as follows: <8 ng/mL; 8–12 ng/mL; and >12 ng/mL. (a) Incidence rate of DGF by tacrolimus concentration. (b) Incidence rate of AR by tacrolimus concentration. ^∗∗^*p* < 0.01 for tacrolimus concentration < 8 ng/mL *VS*. 8 ng/mL < tacrolimus concentration < 12 ng/mL. ^#^*p* < 0.05 for tacrolimus concentration < 8 ng/mL*VS*. tacrolimus concentration > 12 ng/mL.

DGF occurred in 29 (11.3%) of 256 patients during the first week after transplantation. The probability of DGF at different concentration ranges is shown in Figure 3(a). DGF occurred in 10.8%, 6.8%, and 15.8% of patients in the <8 ng/mL, 8-12 ng/mL, and >12 ng/mL groups, respectively, but the association was nonsignificant. AR was identified in 42 (16.4%) of 256 patients during the first postoperative year. The probability of AR at different concentration range is shown in Figure 3(b). We additionally evaluated the effects of immunosuppressive drugs on AR (Table S2). AR occurred in 23.3%, 8.5%, and 11.7% of patients in the <8 ng/mL, 8-12 ng/mL, and >12 ng/mL groups, respectively. Importantly, a clear association was observed between early tacrolimus concentration and AR in our analysis, but not between induction therapy and AR.

## 4. Discussion

The standard method of tacrolimus dosing after kidney transplantation is mainly “one size fits all,” which is subsequently optimized and individualized based on therapeutic drug monitoring. However, tacrolimus shows unpredictable pharmacokinetic in early postoperative kidney transplantation patients, and the “one size fits all” regimen often fails to guarantee clinical efficacy and safety. Identification of the potential factors affecting tacrolimus PK may therefore aid in optimizing individualized regimens for kidney transplant patients. In this study, the CYP3A5 genotype showed a significant association with tacrolimus *C*_0_/*D*. Specific clinical variables (RBC, ALB, and WZC) could also explain residual tacrolimus PK variability. Moreover, the tacrolimus concentration was not clearly associated with DGF but had significant correlation with AR. Our collective results seem to be relevant to a better individualization of tacrolimus regimen that ideally should combine TDM with clinical and pharmacogenetic information in the early postoperative kidney transplant.

Tacrolimus metabolism is mainly mediated by the CYP3A5 enzyme, and many studies have confirmed that CYP3A5 genotype could predict tacrolimus metabolism [8]. In a recent kidney transplantation study, a CYP3A5-guided dosing regimen did not increase the number of patients having therapeutic tacrolimus exposure in the early transplantation period and does not lead to improve acute rejection [12]. Our results indicate that the CYP3A5 genotype is closely related to tacrolimus *C*_0_/*D*. Additionally, ABCB1 (rs1045642 and rs2032582) and ABCC2 (rs3740066) were found to have potential independent effects on early tacrolimus *C*_0_/*D* in multivariate analysis. Previous studies suggested that the CYP3A5 genotype accounts for 50% of tacrolimus PK variability [8, 10]. However, the current study findings indicated a relatively low contribution, which could be attributed to our focus on the immediate postoperative period after kidney transplantation. The perioperative period, especially within 2 weeks after kidney transplantation, is the key time for renal function recovery. During this period, there may be various internal and surgical complications, hemodynamic instability, and pathophysiological manifestations [25–27], which has a strong impact on tacrolimus PK. Accordingly, we speculate that other clinical factors may explain residual tacrolimus PK variability in the early stage after kidney transplant.

We additionally investigated the clinical factors affecting early tacrolimus *C*_0_/*D*. Tacrolimus is mainly confined to RBCs [28] and highly bound to plasma proteins, mainly serum ALB [29], which affect the tacrolimus concentration in circulation. Our results showed that RBC and ALB were associated with a 22.17% and 2.19% increase in tacrolimus *C*_0_/*D*, respectively. The traditional Chinese medicine WZC is a prescription drug (registration number in China: Z20025766), widely used to increase the tacrolimus concentration in solid organ transplantation [30, 31]. Patients who received WZC in our study had 27.2% higher tacrolimus *C*_0_/*D*. Results obtained using the multivariable mixed effects model result indicated that the CYP3A5 genotype only accounted for 16.3% of tacrolimus *C*_0_/*D* variation, while a model combining clinical factors and genotypes explained 43.4% *C*_0_/*D* variability, which was clinically significant increased. Therefore, these findings supported the development of effective tacrolimus dosing regimens for kidney transplant recipients in the perioperative period with combination of genotype and specific clinical variables.

The tacrolimus dosing adjustment regimen in the early period mainly depends on whether the concentration has a negative impact on clinical outcomes of patients. DGF is a significant problem for early allograft survival as it is compounded by acute rejection and allograft nephropathy in the early postoperative period [32]. DGF is an important mediator in the association of tacrolimus metabolism with posttransplant estimated glomerular filtration rate (eGFR), especially in the early period following transplantation [19]. During the transitional period of dialysis, calcineurin inhibitors require low-dose administration, which may increase risk of rejection [33]. The decreased tacrolimus concentration in the early postoperative period may increase the incidence of AR. In clinical practice, it is necessary to maintain a high concentration of tacrolimus after transplantation to prevent rejection. However, the therapeutic levels of tacrolimus in the first week after transplantation may be less important in a combined induction therapy, such as the use of antithymocyte globulin and high-dose steroids at our center. In our study, tacrolimus exposure during the early postoperative period was not associated with DGF, but significantly correlated with AR.

Our research has a number of limitations that should be taken into consideration. Firstly, this is a single-center retrospective design, and multicenter studies are required to validate our results, such as exclusion of the effects of immunosuppressive regimens at different research sites. Furthermore, the tacrolimus concentration is not measured every day in our hospital, especially at weekends, and therefore, some changes may have been overlooked. While the long-term tacrolimus concentration is significantly related to AR, we mainly focused on its association with early tacrolimus concentration in this study, without considering the long-term relationship. Thirdly, we mainly focus on DGF and AR in this study and did not involve de novo diabetes, trembling, or other toxicities, which require further evaluation. Finally, we did not investigate the effects of surgical and donor kidney factors, such as donor-specific antibody (DSA), human leukocyte antigen (HLA), and preoperative panel-reactive antibody (PRA).

## Figures and Tables

**Table 1:** Demographic characteristics of kidney transplant recipients and donors.

Recipients	
Gender (male)	178 (69.5)
Age (years), median (IQR)	41 (34-50)
BMI, median (IQR)	22.59 (20.21-24.82)
Postoperative day (IQR)	16 (10-32)
Comorbidity, *n* (%)	
Hypertension	187 (73.0)
Anemia	117 (45.7)
Hepatitis B	14 (5.5)
Diabetes	12 (4.7)
Anterolisthesis	6 (2.3)
Coronary heart disease	4 (1.6)
Others Laboratory findings^a^, median (IQR)	4 (1.6)
White blood cell count (×10^9^/L)	6.92 (5.2-9.03)
Red blood cell count (×10^9^/L)	3.02 (2.59-3.53)
Hemoglobin (g/L)	91.0 (79.0-105.0)
Hematocrit (%)	27.2 (23.5-32.0)
Platelet count (×10^9^/L)	179.0 (142.0-230.0)
Neutrophil count (×10^9^/L)	5.34 (3.87-7.24)
Total bilirubin (*μ*mol/L)	8.4 (6.5-11.17)
ALT (U/L)	26.0 (17.0-37.0)
AST (U/L)	16.0 (13.0-20.0)
ALP (U/L)	62.0 (47.0-82.0)
TP (g/L)	62.1 (57.02-69)
ALB (g/L)	39.0 (34.9-43.5)
BUN (mmol/L)	10.84 (7.62-16.97)
Cre (*μ*mol/L)	835.3 (700.75-916.78)
Uric acid (*μ*mol/L)	325.1 (253.45-411.6)
Immunosuppression regimens	
Induction agent, *n* (%)	
Basiliximab	32 (12.5)
Antithymocyte globulin	224 (87.5)
Antiproliferative agent, *n* (%)	
Mycophenolate	239 (93.4)
Azathioprine	17 (6.6)
Donors	
Gender (male)	175 (68.4)
Age (years), median (IQR)	49 (37-59)
BMI, median (IQR)	24.2 (21.5-29.6)

^a^The first day after kidney transplantation. BMI: body mass index; ALT: alanine aminotransferase; AST: aspartate aminotransferase; ALP: alkaline phosphatase; TP: total protein; ALB: albumin; BUN: blood urea nitrogen; Cre: creatinine.

**Table 2:** Multivariable mixed effects model for tacrolimus concentration/dose ratio (*C*_0_/*D*).

Variable	Percentage change in *C*_0_/*D* (95% CI)^a^
Anemia	-4.01 (-10.96–3.48)
Postoperative day	0.04 (0.02–0.06)^∗^
RBC^b^	22.17 (14.98–29.82)^∗∗^
HGB	0.07 (-0.10–0.24)
ALB	2.19 (1.72–2.67)^∗∗^
WZC	27.2 (19.39–35.52)^∗∗^
CYP3A5 genotype (rs776746)^c^	
Poor metabolizers	Reference
Intermediate metabolizers	-22.39 (-27.82 to -16.56)^∗∗^
Extensive metabolizers	-40.89 (-47.62 to -33.29)^∗∗^
ABCB1 (rs1128503)	
TT	Reference
CT	-5.21 (-12.07–2.18)
CC	-11.43 (-20.11 to -1.81)
ABCB1 (rs2032582)	
GG	Reference
GA	8.19 (-0.52–17.66)
AA	16.52 (4.11–30.42)^∗∗^
ABCB1 (rs1045642)	
TT	Reference
CT	-14.59 (-23.18 to -5.03)^∗∗^
CC	-15.03 (-24.75 to -4.05)^∗∗^
ABCC2 (rs2273697)	
GG	Reference
GA	1.97 (-22.92–24.65)
AA	8.98 (-13.14–36.73)
ABCC2 (rs3740066)^d^	
CC	Reference
CT	5.59 (-7.32–20.29)
TT	14.42 (0.48–30.29)^∗^
POR28 (rs1057868)	
CC	Reference
CT	-5.41 (-14.41–4.53)
TT	-7.54 (-16.56–2.46)
PXR (rs6785049)	
GG	Reference
GA	-6.12 (-15.82–4.68)
AA	-5.90 (-15.22–4.43)

RBC: red blood cell; HGB: Hemoglobin; ALT: alanine aminotransferase; ALB: albumin; WZC: Wuzhi capsule. ^a^Analysis based on log-transformed *C*_0_/*D*. Model coefficients were exponentiated to provide the percentage change in *C*_0_/*D* for a one-unit change in each covariate, unless otherwise specified. Increases in *C*_0_/*D* signify decreased tacrolimus clearance. ^b^Serious collinearity between RBC and HCT was observed, and consequently only RBC was retained. ^c^Poor metabolizers defined as CYP3A5∗3/∗3 (GG); intermediate metabolizers defined as CYP3A5∗1∗3 (AG); extensive metabolizers defined as CYP3A5∗1∗1 (AA). ^d^Serious collinearity between ABCC2 (rs3740066) and ABCC2 (rs717620) was observed, and consequently, only ABCC2 (rs3740066) was retained. ^∗^*p* < 0.05;  ^∗∗^*p* < 0.01.

## Data Availability

The data and materials during the current study are available from the corresponding author Yu Zhang (zhangwkp@163.com) on reasonable request.

## Additional Points

The authors have completed the STROBE reporting checklist.

## Supplementary Materials

Supplementary Materials Table S1: univariate analysis of tacrolimus concentration/dose ratio (*C*_0_/*D*). Table S2: effects of immunosuppressive drugs on acute rejection (AR). Figure S1: pharmacogenetic analysis of ABCB1, ABCC2, POR28, and PXR polymorphism.
