# Supplementary material for: Tacrolimus Concentration Is Effectively Predicted Using Combined Clinical and Genetic Factors in the Perioperative Period of Kidney Transplantation and Associated with Acute Rejection
Source: J Immunol Res. 2022 Sep 9;2022:3129389. doi: 10.1155/2022/3129389 (PMC9481373; doi:10.1155/2022/3129389)
Supplement: Supplementary Materials — Table S1: univariate analysis of tacrolimus concentration/dose ratio (C0/D). Table S2: effects of immunosuppressive drugs on acute rejection (AR). Figure S1: pharmacogenetic analysis of ABCB1, ABCC2, POR28, and PXR polymorphism. [file 3129389.f1.docx]

Table S1. Univariate analysis of tacrolimus concentration/dose ratio (C_0_/D)

| Variable | Beta | P.value |
| --- | --- | --- |
| Sex (female) | -0.004 | 0.936 |
| Age | -0.002 | 0.486 |
| BMI | -0.002 | 0.789 |
| Postoperative day | 0.001 | <0.001 |
| WZC | 0.276 | <0.001 |
| **Comorbidity** |  |  |
| Hypertension | -0.36 | 0.935 |
| Anemia | -0.254 | <0.001 |
| Diabetes | 0.073 | 0.573 |
| Hepatitis B | -0.263 | 0.09 |
| Coronary heart disease | 0.058 | 0.751 |
| Arthrolithiasis | -0.029 | 0.851 |
| Others | -0.119 | 0.478 |
| **Laboratory findings** |  |  |
| White blood cell counts | -0.022 | 0.361 |
| Red blood cell counts | 0.341 | <0.001 |
| Hemoglobin | 0.009 | <0.001 |
| Hematocrit | 0.038 | <0.001 |
| Platelet counts | 0.001 | 0.429 |
| Neutrophil counts | -0.004 | 0.412 |
| Total bilirubin | -0.002 | 0.522 |
| ALT | -0.024 | 0.453 |
| AST | -0.001 | 0.68 |
| ALP | 0.003 | 0.531 |
| TP | 0.006 | 0.627 |
| ALB | 0.039 | <0.001 |
| BUN | 0 | 0.195 |
| Cre | -0.001 | 0.226 |
| Uric acid | 0 | 0.625 |
| **Genotype** |  |  |
| CYP3A5 genotype (rs776746) |  |  |
| Poor metabolizers | ref | ref |
| Intermediate metabolizers | -0.27 | <0.001 |
| Extensive metabolizers | -0.352 | <0.001 |
| ABCB1 (rs1128503) |  |  |
| CC | ref | ref |
| CT | 0.103 | 0.101 |
| TT | 0.014 | 0.766 |
| ABCB1 (rs2032582) |  |  |
| GG | ref | ref |
| GA | 0.003 | 0.951 |
| AA | 0.06 | 0.329 |
| ABCB1 (rs1045642) |  |  |
| TT | ref | ref |
| CT | -0.064 | 0.326 |
| CC | -0.058 | 0.367 |
| ABCC2 (rs2273697) |  |  |
| GG | ref | ref |
| GA | 0.218 | 0.005 |
| AA | 0.145 | 0.064 |
| ABCC2 (rs717620) |  |  |
| CC | ref | ref |
| CT | 0.159 | 0.2 |
| TT | 0.122 | 0.355 |
| ABCC2 (rs3740066) |  |  |
| CC | ref | ref |
| CT | 0.154 | 0.043 |
| TT | 0.098 | 0.195 |
| POR28 (rs1057868) |  |  |
| CC | ref | ref |
| CT | 0.054 | 0.396 |
| TT | 0.048 | 0.469 |
| PXR (rs6785049) |  |  |
| GG | ref | ref |
| GA | -0.002 | 0.981 |
| AA | -0.016 | 0.813 |

Abbreviations: BMI, body mass index; WZC, Wuzhi capsule; ALT, alanine aminotransferase; AST, aspartate aminotransferase; ALP, alkaline phosphatase; TP, total protein; ALB, albumin; BUN, blood urea nitrogen; Cre, creatinine

**Table S2** Effects of immunosuppressive drugs on acute rejection (AR).

|  | N% |  |  |
| --- | --- | --- | --- |
|  | Non-AR (n=214) | AR (n=42) | p |
| **Immunosuppression regimens**  Induction agent, n (%)  Basiliximab  Antithymocyte globulin  Antiproliferative agent, n (%)  Mycophenolate  Azathioprine  **Tacrolimus blood level, ng/mL^a^** | 27 (12.6)  187 (87.4)  201 (93.9)  13 (6.1) | 5 (11.9)  37 (88.1)  38 (90.5)  4 (9.5) | 0.748  0.543 |
| <8 ng/mL | 92 (43.0) | 28 (66.7) |  |
| 8-12 ng/mL | 54 (25.2) | 5 (11.9) | 0.008 |
| >12 ng/mL | 68 (31.8) | 9 (21.4) | 0.016 |

a: Tacrolimus concentrations were averaged over the entire hospital stay and then classified.


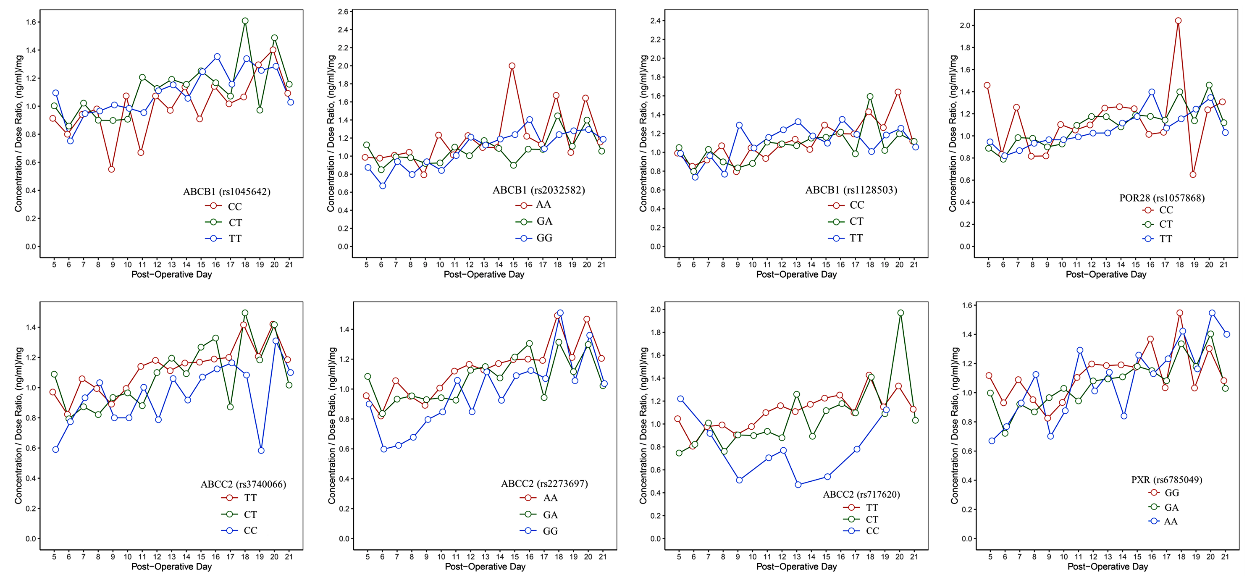


Figure S1. Pharmacogenetic analysis of ABCB1, ABCC2, POR28 and PXR polymorphism.
